# High-throughput cell optoporation system based on Au nanoparticle layers mediated by resonant irradiation for precise and controllable gene delivery

**DOI:** 10.1038/s41598-024-53126-9

**Published:** 2024-02-06

**Authors:** T. E. Pylaev, E. S. Avdeeva, B. N. Khlebtsov, M. V. Lomova, N. G. Khlebtsov

**Affiliations:** 1 Saratov Medical State University N.a. V.I. Razumovsky, 112 Ulitsa Bolshaya Kazachya, Saratov, Russia 410012; 2 Institute of Biochemistry and Physiology of Plants and Microorganisms – Subdivision of the Federal State Budgetary Research Institution Saratov Federal Scientific Centre of the Russian Academy of Sciences, 13 Prospect Entuziastov, Saratov, Russia 410049; 3 Saratov National Research State University, 83 Ulitsa Astrakhanskaya, Saratov, Russia 410012

**Keywords:** Cellular imaging, Cell delivery, Nanoparticles, Nanoparticles

## Abstract

The development of approaches based on genetically modified cells is accompanied by a constant intensive search for new effective and safe delivery systems and the study of existing ones. Recently, we developed a new plasmonic nanoparticle layers-mediated optoporation system that can be proposed for precisely controlled, high-performance laser transfection compatible with broad types of cells and delivered objects of interest. The main goal of the present study is to demonstrate the broad possibilities and advantages of our system for optoporation of several mammalian cells, classified as "easy-to-transfect" cells, namely HeLa and CHO lines, and "hard-to-transfect" cells, namely A431 and RAW 264.7 cells. We show the efficient delivery of various sized cargo molecules: from small molecular dyes propidium iodide (PI) with molecular mass 700 Da, control plasmids (3–10 kb) to fluorophore-labeled dextranes with masses ranging from 10 kDa up to 100 kDa. The performance of optoporation was investigated for two types of laser sources, 800-nm continuous-wave laser, and 1064-nm ns pulsed laser. We provided a comparative study between our system and commercial agent Lipofectamine for transient transfection and stable transfection of HeLa cells with plasmids encoding fluorescent proteins. The quantitative data analysis using flow cytometry, Alamar blue viability assay, and direct fluorescence microscopy revealed higher optoporation efficacy for hard-to-transfect A431 cells and Raw 264.7 cells than lipofection efficacy. Finally, we demonstrated the optoporation performance at the single-cell level by successful delivering PI to the individual CHO cells with revealed high viability for at least 72 h post-irradiation.

## Introduction

Genome editing^1^, immunotherapy^2^, and cell therapy^3^ are promising technologies for developing next-generation approaches for treating and diagnosing socially significant diseases. The listed methods' fundamentals rely on successfully delivering the therapeutic molecules, such as peptides^4^, oligo-^5^ and polynucleotide^6^ DNA and RNA chains, and natural and synthetic organic drugs^7^, to the target cells and tissues. Over the past decade, pilot data have emerged on clinical trials of FDA-approved gene therapy drugs based on molecular complexes and nanosized carriers^8^. Specifically, the gene editing can be performed using two general strategies. The first strategy is implemented by directly administering a therapeutic agent into the host organism, followed by its absorption and expression at the level of target cells^9^. The second strategy is based on the gene modification of target cells isolated from the host organism, multiplication to the needed cell biomass and subsequent re-introduction of edited cells into the host organism^10,11^.

Despite an extensive list of pre-clinical studies^12–14^, several unresolved fundamental problems and serious drawbacks limit the widespread introduction of engineered cell-based technologies into actual biomedical practice. In particular, no single technology still meets all requirements, such as broad compatibility with different cells, sophisticated cost, simplicity, and robustness. The issues of protecting the administered nucleic acid (NA) against natural cellular barriers^15,16^ thus minimizing the geno-^17^ and cytotoxicity^18^ of the carriers and the vectors, remain unresolved. In addition, the existing systems are not compatible with broad types of cells and delivered agents. The lack or limited scalability and automatization,^19,20^ are critical at the stages of transfer to real practice and further commercialization. In this regard, an active search for new delivery systems and the intensive study of the existing techniques based on various bio-^21,21^ or chemically^23^ originated carriers is in high demand. In the last decade, special attention has been paid to developing delivery systems based on various nanoparticles (NP)^24^ and nanostructures^25^ used as carriers and/or mediators of physical action^26^. Carriers and labels based on gold nanoparticles (GNP)^27^ are among the most actively studied and widely used for gene delivery^28^, bioimaging^29^, sensing^30^, photothermal/photodynamic theranostics^31,32^ and other applications. The attractiveness of GNP for biomedical applications is due to the combination of unique optical and geometric parameters^33^.

The extremely popular are delivery systems based on multifunctional micro- and nanocarriers mediated by mechanical forces^34,35^ or various sources of electromagnetic fields^36,37^. One of the promising techniques is delivery mediated by light exposure to a laser source, also called optoporation, optoinjection, or laser infection. There are three main types of optoporation approaches. One of them, so-called direct photoporation^38,39^ is performed by narrow laser beam radiation focused at the cell membrane surface. Unfortunately, the use of femto- or picosecond laser systems is limited due to the narrow range of operating parameters of irradiation with minimal impacts on bio-objects. The other type is optoporation mediated by photosensitizing NP^40–42^, which effectively absorbs laser radiation energy and causes local damage to the lipid bilayer due to photothermal effects^35^. The drawback of such an approach is labored control and management of the effect of NP-mediated laser irradiation caused by nonspecific adsorption of NPs on the cell surface, with possible free penetration of particles into the cytosol^43^. The investigations of the possible adverse effects of NPs on the cells are required, which also imposes appropriate restrictions for subsequent transfer to actual practice. Finally, the third approach is based on the photothermal effects of photosensitive substrates^44^ activated by resonant laser irradiation^45^. The advantageous features of the last strategy among others are as follows. First, the fine adaptation of irradiation regimes and substrate parameters to the individual morphophysiological characteristics of cells opens up possibilities for expanding the list of compatible cell types and delivered agents. Second, using chemically and physically stable substrates completely excludes the direct effect of NP on adherent cells and their penetration into cells. Third, the absence of an additional incubation step compared to NP suspensions dramatically simplifies the procedure and reduces the impact on cell viability. In the last decade, several optoporation systems^46–49^, implemented on photoactive substrates were developed. However, most of the established techniques are pretty laborious, expensive, poorly scalable, thus need further investigations prior to be transfered to the real practice. As far as we concerned, only few technologies are commercialized to the date, e.g. the pyramid-like substrates-based optoporation system developed by the E. Mazur’s group^50^.

We recently developed the plasmonic-induced cell optoporation system^51^ based on GNP layers for efficient and safe NA delivery and investigated its operating parameters and underlying mechanisms^52^. The GNP layers can be prepared directly on conventional culture surfaces, such as bottoms of polystyrene multiwell plates, Petri dishes, or glass slides^53^, with high uniformity and desired 2D particle density. Among all tested optoporation conditions and GNP properties, the 24-well plate layers formed by Au nanostars (AuNS) with plasmon resonance (PR) peak near 800 nm and surface density equal to 15 μg Au per cm^2^ showed the best optoporation performance in terms of highest NA delivery efficacy, and cell viability level. Thus, this platform is used in the present work.

The main goal of the paper is to demonstrate the wide possibilities and advantages of our system for optoporation of several mammalian cells, classified as "easy-to-transfect" cells, namely HeLa and CHO lines, and "hard-to-transfect" cells, namely A431 and RAW 264.7 cells. We show the efficient delivery of various sized cargo molecules: from small molecular dyes (propidium iodide (PI), 700 Da), control plasmids (3–10 kb) to fluorophore-labeled dextranes with molecular weight ranging from 10 kDa up to 100 kDa. We demonstrate the capabilities of the system on two fundamentally different types of irradiation. The first one is a continuous-wave (CW) laser at a wavelength of 800 nm using a portable setup with a defocused beam over the entire diameter of the irradiated surface. The second is a pulsed nanosecond laser with a source wavelength of 1064 nm, a narrow-focused beam of 3 μm, and an automated X–Y scanator. The performance of GNP layers-mediated optoporation for transient and stable transfection with plasmid DNA is compared with the commercial agent Lipofectamine 2000 (LF)^54^. In addition, we show the possibility of optoporation on rare cell monolayers and at the individual cell level by fine-tuning the irradiation conditions. The cell viability and delivery efficacy are analyzed using flow cytometry (FACS), Alamar blue viability test, luciferase luminescent assay, laser confocal scanning microscopy (CLSM) imaging, and direct microscopy observation in phase-contrast and fluorescence modes.

## Materials and methods

The reagents and materials are listed in Section S1, Supporting Information. The step-by-step procedure, namely the AuNS synthesis and layer fabrication, cell cultivation, cell viability assessment, and the analytical measurements of optoporation results, are described below and demonstrated in the Supplementary video file.

### Preparation and characterization of GNP layers

According to our previously developed technology^53^, AuNS layers with Au surface density equal to 15 µg/cm^2^ were prepared on the bottoms of the 24-well plates. The AuNS colloid with a PR band near 800 nm was fabricated by the two-stage seeding method proposed by Yuan et al.^55^ with our minor modifications^56^. The extinction spectra of as-prepared AuNS were recorded on a Specord S-300 Vis spectrophotometer (Analytik Jena, Germany). The average hydrodynamic particle diameter, size distribution, and zeta potential were obtained using a Zetasizer Nano ZS dynamic light scattering (DLS) analyzer (Malvern, UK). The particle morphology was characterized by the transmission electron microscopy on a Libra-120 TEM microscope (Carl Zeiss, Germany) at the Center for Collective Use "Symbiosis" of the Institute of Biochemistry and Physiology of Plants and Microorganisms, Russian Academy of Sciences.

To fabricate the layers, the bottom of the wells of 24-well polystyrene culture plates (Corning Costar, USA, GEB, China) was initially treated with 1% polyvinyl pyridine (PVP) ethanol solution for 2 h at room temperature. The volume of the PVP solution was 250 μl, just to cover the bottom of the wells. After incubation, the PVP solution was decanted, and the wells were washed thrice with 96% ethanol and Milli-Q deionized water (Merck Millipore, Germany) to remove excess PVP. Next, 800 μl of AuNS colloid with an optical density 1 (as measured in a cuvette with a 1 cm optical path) at 800 nm was added to each well. Then, the plates were CF at 1000 g for 5 min in an Eppendorf 5810R centrifuge (Eppendorf, Germany) equipped with an S-4–104 plate-bucket rotor. The supernatant was decanted, and the wells were washed with water and air-dried. The electron micrographs of the layers were obtained using a Mira SEM microscope (Tescan, USA). The degree of particle adsorption and the 2-D packing density in each well was estimated by integral subtraction of the extinction spectra of freshly prepared AuNS layers and supernatants after CF, measured on a Spark 10 M plate UV–vis multifunctional reader (Tecan, Austria), as described in details in our previous study^52^. Coating uniformity was assessed by measuring the optical density of the plates with layers in a 2D scanning mode with 100 × 100 μm mesh resolution on a Spark 10 M reader at a fixed wavelength of 800 nm, corresponding to the AuNS PR peak. Finally, the plates with AuNS coatings were sterilized by incubation in 96% ethanol for 15–30 min with subsequent UV treatment for 30 min and stored at 4 °C for further optoporation experiments.

### *The AuNS mesh gridding *via* laser engraving*

The coordinate grid was applied to the AuNS layers by laser engraving using the LSF 20H nanosecond laser machine (HGTECH, China), with automatic movement in the X–Y coordinates along the uploaded trajectory. The coordinate grid was set inside a 2 × 2 mm square divided into 100 × 100 µm subdivisions, additionally marked horizontally with A to T letters and vertically from 1 to 20 numbers. Similarly, the coordinate grid was prepared on glass substrates in a recent work^57^. The engraving was carried out by completely "burning out" the NPs from the plastic surface under the laser exposure with the following regimes: pulse energy 7 μJ, frequency 39 kHz, pulse duration 200 ns, scanning speed 231 mm/s. After single irradiation, the engraved AuNS were removed by rinsing the wells with distilled water.

### Cells cultivation

The cell lines used in the experiments were as follows: human cervical carcinoma HeLa cells; human epidermal carcinoma A431cells; monocyte-macrophage-like RAW 264.7 cells derived from a cell line transformed with Abelson leukemia virus derived from BALB/c mice; and Chinese hamster ovarian CHO epithelial cells. All cell lines were obtained from the Russian Collection of Cell Cultures (Institute of Cytology, Russian Academy of Sciences, St. Petersburg). The cells were harvested in culture flasks and supplemented with full DMEM medium containing 10% fetal bovine serum and a mixture of penicillin–streptomycin-neomycin antibiotics with 100 µg/mL concentrations. The cell medium was refreshed every 3–5 days. The cells were maintained in the incubator (Innova CO-14, New Brunswick Scientific, Germany) at a temperature of 37 °C in the humidified atmosphere with 5% CO_2_. The cells were passaged at 80–90% monolayer confluence by detaching from the surface via trypsin treatment for 5 min. Before 48 h to the experiments, the cells were seeded in the sterilized 24-well plates with AuNS layers or on the pure plastic (for controls). The seeding dose was 2*10^4^ cells per well to achieve the initial confluency of about 70% at the start of the optoporation. The seeding dose was estimated using an automatic cell counter TC20 (BioRad, USA). All cell lines were checked for the absence of mycoplasma contamination by PCR assay using a certified kit according to the manufacturer's instructions (Evrogen, Russia). The experiments were conducted between the second and 10th passages.

### Laser sources

The optoporation in a CW mode was performed using a hand-made setup with an 808-nm laser source with a nominal power of 4 W (Optronics, Russia) and a defocused beam across the entire well. The optoporation in a pulsed mode was performed using the commercial system LSF-20H (HGTECH, China) with an ytterbium 200 ns laser source with 1064 nm wavelength, nominal power of 20 W, with a narrow-focused beam of about 3 µm and automatic displacement along the X–Y-Z axis with adjustable trajectory and ranging scanning speed. The irradiation modes for the performed experiments are given in the subsequent sections within the description of the corresponding results.

### Optoporation of the cells on AuNS layers for the delivery of fluorescent agents and transient transfection of plasmid DNA

The typical optoporation experiment included the following steps: the cultivation of the cells in the wells with AuNS layers up to 70% confluency; the replacement of the medium with a serum-free DMEM medium before irradiation; single laser irradiation; the addition of the delivered agents into the culture medium in appropriate concentrations (15 µM for PI dye and 500 ng/mL for pDNAs); incubation for 6 h with subsequent medium replacement by the complete DMEM; the determination of transfection efficacy as well as cell viability monitoring at all stages of the experiment. The characteristics of all used cargo molecules and pDNAs are given in the S3, Supporting information. The experimental sets included the following sample and control wells: optotransfection with the pulsed laser (sample), optotransfection with the CW laser (sample), lipofection (commercial control), cells with added pDNA without any treatment (free delivery control), intact cells without any treatment (blank). The lipofection was performed according to the manufacturer's recommendations.^54^ The experimental details are described below in the relevant subsections of Results and Discussion.

### *Producing the HeLa cells with stable expression of fluorescent proteins *via* optoporation*

The stable integration of pDNA in the host cell genome to obtain constitutive expression of intracellular mCherry protein or extracellular luciferase pGLuc was performed following the standard clone-selection procedure^58^. At the initial stage, the semi-lethal dose (LC_50_) of the selective antibiotic geneticin (G418) was determined by cultivating the HeLa cells with DMEM medium containing the G418 solution in the concentration range from 0 to 1400 μg/mL. The experimental set included the following wells: cells after successful transient transfection via the pulsed laser optoporation (sample 1) or with the CW laser (sample 2); cells after successful transient transfection via lipofection (commercial control), cells with added pDNA without any treatment (free delivery control). The HeLa cells, baring pDNA, were treated with the selective DMEM medium containing G418 in LC_50_ doze, starting at 96 h post optoporation or lipofection, respectively. The selective medium was refreshed every 3 days, by reaching 80% monolayer confluency the cells were passaged to the new wells. The cells with no selective antibiotic added to the medium, were harvested analogous to all sample ones, and served as a growth control. Antibiotic-resistant clones were selected for at least 3–4 weeks until the complete monolayer of cells expressing the fluorescent protein was obtained. The number of mCherry^+^ cells was counted by processing the confocal microscopy images, and the level of Gluc was measured by luciferase assay. Finally, the modified HeLa cells were cryo-frozen and stored in the liquid nitrogen tank for 3 months. Subsequently, the samples were defrozen, harvested to complete monolayer in a culture 25-cm^2^ flask, and subjected to the quantification of fluorescent-tagged cells.

### The transfection efficacy quantification

*Direct fluorescence microscopy for PI visualization.* Direct fluorescence microscopy was used to evaluate the efficiency of PI delivery to the cells. Bright-field and fluorescent images were obtained using I3, N21, and D light filters of an inverted DMI3000 microscope with transmitted-light illumination system, under various magnifications with 5x, 10x, and 20 × objectives, and captured by high-sensitivity 420D CCD camera (Leica Microsystems, Germany). The relative number of PI^+^ cells after irradiation (sample) normalized to the average number of cells in the monolayer (without exposure, control) was determined by processing fluorescent microscopic images. At least 1000 cells were taken from each sample for registration.

*FACS analysis* was used to quantify the optotransfected cells expressing GFP and GLuc. The measurements were conducted on an Amnis® Imaging Instrument Comparison cytometer (Luminex, USA). The sample preparation procedure included the following steps: trypsinization of the cells 72 h after transfection; triple CF and sediment dispersion in an equivalent volume of phosphate-buffered saline; concentration of the cell sediment after the third wash in 30 µl of calcium and magnesium ions-free phosphate buffer for maximum cell disaggregation. Next, the samples were subjected to cytofluorimetric analysis in the following modes: λ_ex_/Δλ_em_ = 488/500–560 nm, with an excitation laser power of 200 mW. At least 4 repeated analyses of each sample were conducted with no less than 4,000 cells per measurement. The FACS data post-processing was performed according to the following algorithm: the objects that had normal spherical shape and size corresponding to living cells were selected for analysis; the dead, fragmented cells and debris were cut off; the autofluorescence threshold was determined from the control cells; finally the positively fluorescent cells were counted as the number of objects standing above the autofluorescence threshold. The analysis for each sample (cells detached from single well) was conducted in triplicates with no less than 10,000 cells per measurement.

*Assessment of luciferase activity.* The luciferase level indicating the efficacy of cell transfection with the pGluc plasmid was measured using the commercial kit (NEB, Netherlands) following the manufacturer's instructions. Briefly, 200 μL of the culture medium was collected for analysis at 48, 72, and 96 h time points from optoporation. The luminescence reaction was initiated by mixing 10 µl of samples with 50 µl of a commercial luciferin solution in 96-well microplates with clear bottoms and darkened wells. The luminescence intensity was recorded immediately after mixing all components, using a Cary Eclipse microplate reader (Agilent Technologies, USA) with a wholly closed excitation channel, λ_em_ = 475 nm, integration time 5 s.

*CLSM imaging for pDNA visualization*. The fixed cell samples were prepared by standard procedure.^59^ Briefly, the cells were grown on coverslips with all subsequent optoporation steps described above without any modifications. 72 h post-transfection, the wells with coverslips were treated with 3.6% formaldehyde solution for 2 h at room temperature (RT); then washed with PBS five times and treated with DAPI solution (100 ng/ml) to counterstain the cell nuclei for 30 min at RT in the dark; three times washed with a PBS solution to remove excess DAPI solution. Finally, the coverslips were transferred to a glass slide and mounted with the addition of an anti-fade reagent to provoke photobleaching and autofluorescence. The fluorescent images were captured on a TCS SP8 X confocal laser scanning microscope with a 40 × objective (Leica Microsystems, Germany). The excitation and emission wavelengths were adjusted for GFP, Gluc, or RFP signal detection. The relative number of fluorescence optoporated cells normalized to the total number of cells in the monolayer was determined by processing the CLSM images. At least 5000 cells from each sample were taken for analysis.

### Cell viability assays

Cell viability at different stages of the experiment was determined using a standard analysis of cell metabolism, namely the resazurin test, and by direct microscopic observation of cell morphology and the state of the monolayer at various magnifications. According to the manufacturer's recommendations, the resazurin assay was performed using the commercial Alamar blue reagent.^60^ The cell medium was replaced with a resazurin solution in full DMEM at a 50 μg/mL concentration. The plates were then incubated for 72 h at 37 °C in the presence of 5% CO_2_. At several time points, namely, 3, 5, 24, 48, and 72 h post addition of Alamar blue, 100 μl of the medium was taken for fluorometric analysis. The measurements were performed on a Cary Eclipse microplate reader (Agilent Technologies, USA) with the excitation wavelength 530 nm, emission wavelength 600 nm, and 10 nm slit width. The fluorescence intensity values in the experimental and control wells were normalized to the negative control (resazurin solution in a DMEM medium).

### Statistical data processing

Fluorescence microscopy images were analyzed using ImageJ open-source software. FACS data were analyzed using certified Amnis software supplied with the cytometer. All experimental sets were conducted in independent triplicates, with no less than 50 wells of each control and sample types (specified individually in appropriate subsections) per experiment, respectively. The controls were as follows: intact cells without any treatment, cells grown on the AuNS layer, with or without cargoes incubation and with or without laser illumination. All obtained data was processed using the MS Excel program using the Student's Criteria t-test. The average values of each data are presented in the graphs and histograms, and the standard deviations at a significance level of *p* < 0.05 are set for error bars.

## Results and discussion

### Tuning the AuNS layers parameters for best optoporation performance

Since this study aims to demonstrate the feasibility of our developed optotransfection system on various cell models, an important issue is the selection of the most convenient plasmonic layers parameters and sophisticated cell format for further data analysis. In our recent study^52^ we provided comparative analysis of GNP layers with different particle geometry and plasmonic properties, and the AuNS layers with a PR at 800 nm were selected among all tested due to the beneficial technical features of their fabrication and the highest values of optoporation performance. Thus, we used this platform in the present study for all experiments.

The as-prepared AuNS have average hydrodynamic particle size 82 ± 7 nm and Zeta potential -21 ± 3.2 mV (measured by DLS), and extinction PR peak at the wavelength of 800 nm (Fig. S1, Section S3, Supporting information) that allows efficient cell optoporation in a resonant mode using an 800-nm laser source. Remarkable, that recently we explored that AuNS present another resonance mode near 1100 nm, by performing special spectroscopic measurements confirmed by spectral simulations^61^. Therefore, guided by these findings data, we assume the prospects for AuNS usage for an effective optoporation under 1064-nm irradiation. The issues adressing the precise mechanisms of cell changes under laser irradiation in the on- and off-resonance^62^ modes, are worth to be further investigated outside the framework of this study. It should be mentioned that well-known hypotheses are mainly referred to the heat-shock^39^ and the vapor nanobubbles^40^ concepts, depending on the optoporation regimes.

**Figure 1:**
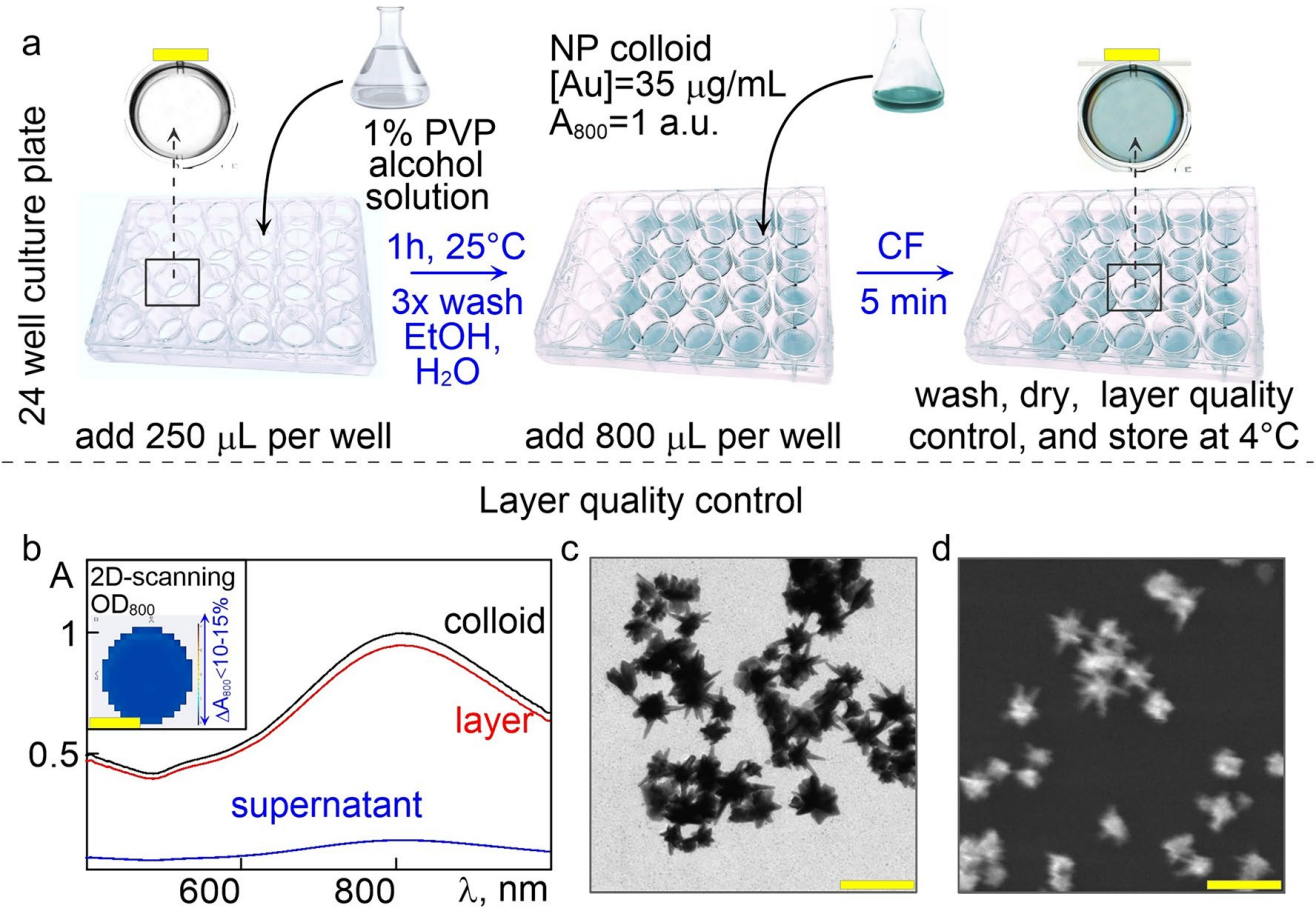
(**a**) Schematic representation of the AuNS layers preparation technology. (**b**) Normalized extinction spectra of the initial colloid, the supernatant after CF, and the obtained layer. The inset shows the spectrograms of the layer's uniformity. (**c**) Typical TEM image of as-prepared AuNS colloid. (**d**) SEM micrographs of AuNS layers. Scale bars correspond to 5 mm (enlarged scanned images of culture plate wells, a, b) and 100 nm (electron micrographs of particles, c, d).

The well-established problem while working with cells with low adherent and proliferation abilities, such as primary or stem-like cells, is obtaining reproducible cell monolayers on sophisticated cultural surfaces with needed confluence before the transfection. Regarding this task, we have fabricated the AuNS layers on the bottoms of the wells of standard 96-, 48-, 24-, 12-, and 6-well culture plates and Petri dishes with a diameter of 35 mm. Among all tested types of plastic, the 24-well plate format with a surface of well equal to 3.8 cm^2^ was selected (Fig. 1a), according to considerations of the needed number of transfected cells for further adequate statistical analysis, thus giving no less than 3 × 10^5^ cells per well.

Another parameter that may impact optoporation performance is the 2D packing density of particles in AuNS layers. However, the direct microscopic observation of the cell monolayers cultured on AuNS layers (see below Figs. 3, 5, 6, 7) is sufficiently complicated with increasing AuNS packing density: the gray-blue color of the layer acts as a darkening filter. Thus, the high-density background from the AuNS layer with a packing density of more than 28 μg/cm^2^ makes the cells hardly visible in the phase-contrast mode. Therefore, we have selected the AuNS layers with a median packing density equal to [Au] ~ 15 µg/cm^2^ for the present experiments.

### Characterization of AuNS layers

The uniformity of particles packing in the layer was determined by recording the optical density of the AuNS layers at a fixed wavelength (λ = 800 nm, corresponding to the PR peak) in the 2D scanning mode over the entire area. The output data was obtained using Tecan software, and the resulting spectrogram is depicted in the inset Fig. 1b. It represents the degree of coating uniformity in terms of the color gradient, which expresses the deviations of the mean value of the optical density on the PR wavelength. Using this quality control check, we used the layers with no more than 5–7% non-uniformity degree for further experiments. The similar extinction spectra of the layer compared to the as-prepared spectra of the AuNS colloid (Fig. 1b) indicate the absence of large aggregates of the particles during the assembly. The unchanged star-like geometry of initial particles, as depicted on the TEM image (Fig. 1c) was revealed by SEM analysis of the AuNS layers (Fig. 1d) and investigated in detail in our recent study^52^. The worth biocompatibility of the AuNS layers for further optoporation experiments, as well as the reagents used for their preparation, was proven by Alamar blue assay (Table S3, Section S5, Supporting information).

The next valuable characteristic is the adsorption value. It was determined by subtracting the normalized optical density values at a wavelength of 800 nm of the initial colloids and supernatants after CF. The packing density of AuNS in the layer was calculated by normalizing the values of the optical density of the initial colloids to the degree of adsorption of particles in the layer, taking into account the considerations of the complete reduction of HAuCl_4_ during the synthesis of AuNS and formation of single particle layer. The details of these calculations are given in our previous study^52^.

**Figure 2:**
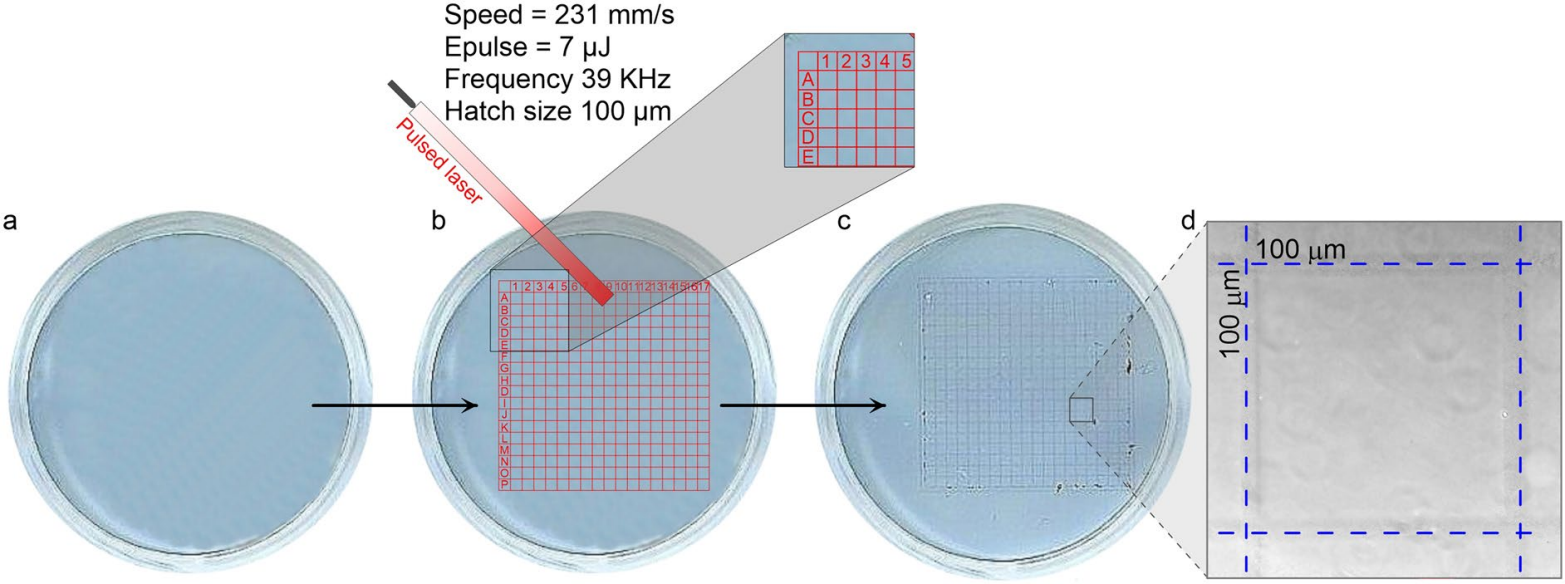
Coordinate mesh gridding via AuNS layer laser engraving. (**a**) Scan image of the AuNS layer on the well bottom of the 24-well plate. (**b**) Schematic representation of laser engraving with set laser parameters. (**c**) Scan image of the grid-marked AuNS layer. (**d**) Phase-contrast micrograph of the gridded AuNS layer, 20 × objective.

To perform the optoporation at the single-cell level and trace the long-term post-effects on the same cells, we marked AuNS layers with coordinate grids. The grids were applied via laser engraving of the AuNS from the layers pulsed laser irradiation (Fig. 2). The coordinate grid was applied on a 2 × 2 mm square, with 100 × 100 μm mesh size, numbered horizontally from A to T and vertically from 1 to 20. The mesh size was chosen to observe single cells with 10x, 20x, and 40 × microscope objectives; see below section 'Optoporation of individual cells'.

### The optoporation experimental setup

**Figure 3:**
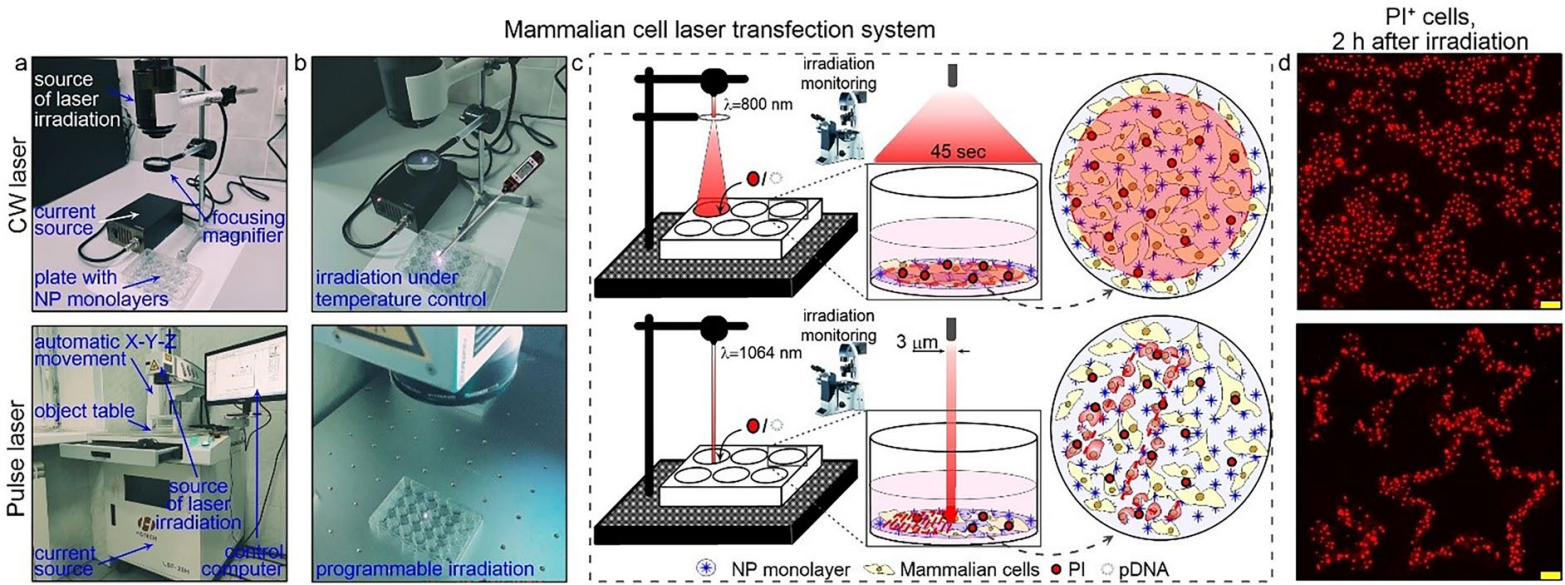
The experimental setup of optoporation systems: with CW laser source (the upper row), and pulsed laser source (the below row). (**a**) Photoimages of the optoporation systems. (**b**) Photoimages of the 24-well plate with AuNS layers under the laser exposure. The red light from the satellite diode visualizes the irradiated well. (**c**) Schematic representation of the experimental setup and the working principle of the optoporation system. (**d**) Fluorescent microscopy images of optoporated HeLa cells with the delivered PI dye. Scale bars correspond to 100 µm.

We used two laser sources for NIR irradiation: CW 808-nm laser and pulsed ns 1064-nm laser. The selection of the sources wavelengths is based on the following general statements: (1) to perform optoporation in a resonance mode the laser wavelength should be fitted to the plasmon bands of the AuNS layers, 800 nm and 1100 nm, respectively; (2) the effects of laser exposure on the cells and the cell medium should be minimized (the absorbance values of DMEM components, such as fetal serum albumin, the red colored pH indicator, are less in the NIR region than that in the visible region). The layout of the experimental setup and images of laser sources are depicted in Fig. 3. The optimal irradiation conditions for HeLa cells with CW laser were determined recently^51^ and used here as default, specifically: irradiation energy 25 J, incident intensity 1 W/cm^2^, exposure area 3.6 cm^2^, and irradiation time 50 s. The optimal irradiation modes for pulsed laser exposure, namely, the pulse energy, the pulse duration, the frequency, and the scanning speed, were determined according to Fig. S2 (Section. S4, Supporting information). Briefly, the cell monolayers grown on AuNS layers were subjected to single irradiation at ranging values of one of the parameters and unchanged values of the others. For each set of ranging irradiation modes, the relative number of PI^+^ cells was counted 30 min post-irradiation and the cell viability was assayed 24 h post-irradiation (Table S2, Section. S4, Supporting information). The estimated optimal modes resulting in maximal levels of optoporation efficacy and cell viability were as follows: pulse energy 1.6–2 µJ, pulse duration 200 ns, pulse frequency 10 kHz, and scanning speed 20 mm/s. Then we performed necessary control experiments with all cell lines used in the study, to assess the effects on cell viability of laser irradiation under selected optimal modes, the effects of cargo molecules and the AuNS layers, separately and in various combinations, respectively. The obtained data (Table S4, Section. S5, Supporting information) revealed non-significant changes in viability for all control sets, thus confirming that used regimes were suitable for further optoporation experiments. By performing additional experiments, we proved effective delivery of fluorescent dyes and dye-labeled dextranes with ranging molecular masses to HeLa cells (Fig. S3, Section S6 Supporting information).

We used a set of plasmid DNA as model delivery agents (Section S2 Supporting information). The genome of all used plasmids included typical elements for integration and expression in host mammalian cells, namely the genes coding fluorescent proteins (GLuc, GFP, mCherry), the cytomegalovirus promoter (pCMV), and an integrated antibiotic resistance cassette (for geneticin, G418). The plasmid DNA (pDNA) was multiplied via a standard clone-selection procedure in the *E. coli* system with subsequent maxi-prep isolation (Section S7, Supporting information).

### Comparative study of optotransfection vs. lipofection for HeLa cells transient transfection with pDNA

**Figure 4:**
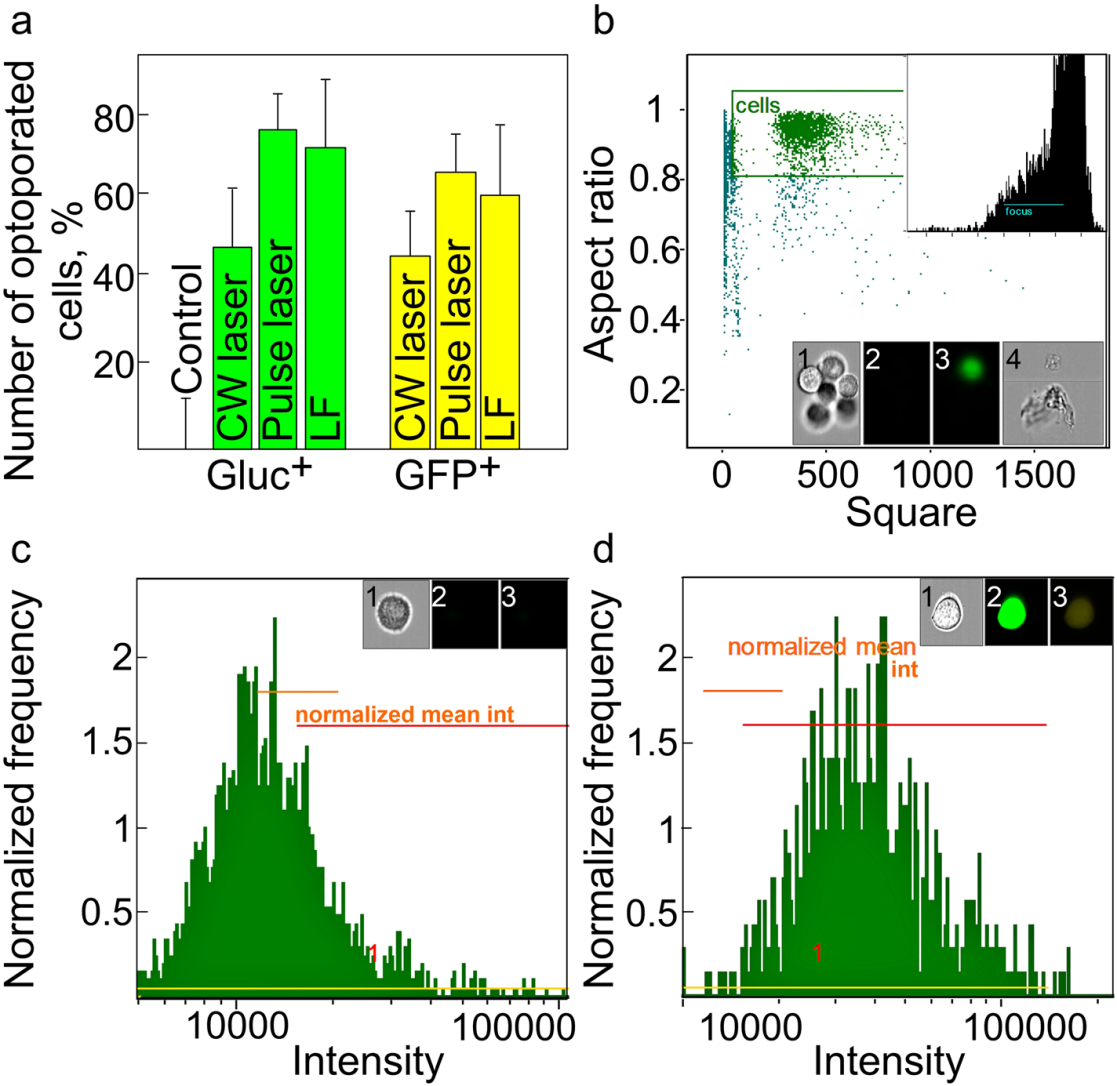
(**a**) Relative number of sample and control HeLa cells expressing Gluc and GFP, determined by FACS 72 h after optotransfection with CW laser, pulsed laser and lipofection with LF. (**b-d**) Algorithm for post-processing of the FACS data. The inset microimages, acquired by AMNIS instrument on three emission channels under 488 nm excitation, depict the cell debris (**b, 1–4**) the cell aggregates (**b**), the control cell (**c**), and experimental cell (**d**) samples taken on three channels of the cytometer **(1, 2, 3).** The mean data is represented in the bars (**a**) with the standard deviations at a significance level of *p* ≤ 0.05 set for the error bars.

The efficacy of optotransfection and lipofection of HeLa cells with pDNA was recorded 72 h post-transfection and quantified using FACS analysis. The representative data are depicted in Fig. 4, namely the final diagram of the processed samples (Fig. 4a), extracted from the scatter plot (Fig. 4b); the defined signal thresholds (Fig. 4c,d) and the microimages of analyzed cells, the cell debris and other artifacts excluded from analysis.

According to the obtained FACS data (Fig. 4a), the efficacy of optotranfection with pulsed laser (72 ± 8% GFP^+^ cells and 63 ± 9% GLuc^+^ cells) was comparable to the lipofection efficacy (69 ± 11% GFP^+^ cells and 58 ± 10% GLuc^+^ cells). However, the irradiation with the pulsed laser, with adequately selected irradiation modes, acts more delicately than the CW laser, thus giving an increased number of transfected and viable cells. The cell viability was quantified 72 h post-transfection using Alamar blue assay, which revealed 90 ± 4% for optotransfection and 75 ± 5% for LF, respectively. Therefore, we can conclude that our optotransfection system can be considered for safe and efficient transfection of pDNA with exceeded performance compared to lipofection.

### Optotransfection for obtaining the HeLa cells with stably expressed fluorescent protein

**Figure 5:**
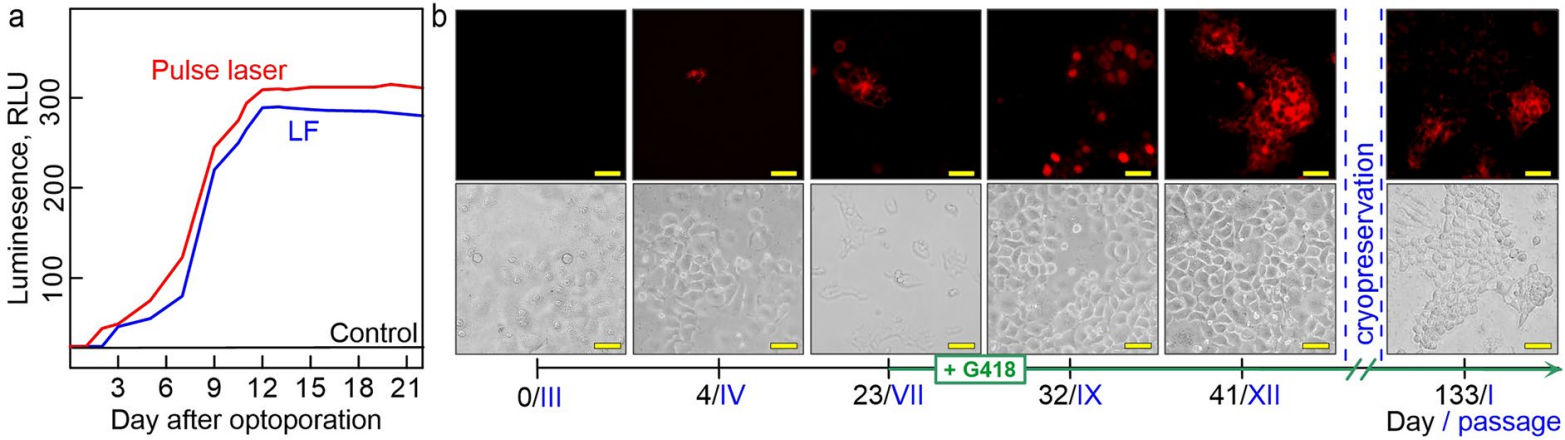
(**a**) Luciferase activity of the optotransfected HeLa-GLuc^+^ cells, lipofected cells, and control cells. (**b**) Representative CLSM microimages of optotransfected HeLa-mCherry + captured throughout the selection process until the complete monolayer with stably transfected cells was obtained; and after the cycle of cryopreservation/depreservation. The days of the experiment are designated below in arabic, the corresponding passages in roman numerals. The scale bars are 50 µm.

To the date, a broad list of laboratory protocols^16^ as well as commercially available services^64^, are available for customized production of cells with long-term constitutively expressed phenotype of interest. However, the unresolved limitations significantly narrow the list of cells compatible with stable transfection. In particular, according to the general recommendations, the efficacy of transient transfected cells taken as “starting material” should be no less than 50%. This requirement is based on the following considerations: (1) a significant part of the cells are lost at the stage of clones selection; (2) the foreign DNA integration into the host genome have unpredictable probability and therefore, is poorly controlled; (3) the proliferation and the adhesion activities of the survived cells in a rarely dense monolayer after clone selection are lowered due to the lack of cell “neighbors” and non-sufficient amount of cell matrix. The next critical parameter for many cell lines is the number of working passages, thus regarding the limits to the operation time (the standard experimental cycle for stable transfection is 3–5 weeks, which is equal to 20 or more passages). Guided by the given considerations, we performed a proof-of-concept comparative study (optotransfection *vs* commercial lipofection) of the stable transfection of HeLa cells with pDNAs with encoded fluorescent proteins genes (mCherry / GLuc). The goal was emphasized to decrease the operating time within increase of the integral transfection efficacy. We conducted the long-term monitoring of the cell viability and quantified the level of intracellular and extracellular expression of fluorescent proteins. Specifically, the estimated lower threshold time point post-transfection at which detectable levels of Gluc expression were achieved by luciferase assay (Fig. 5a), was 2 days for the optotransfected samples, and 3 days for the lipofected ones. The samples with optoporated cells reached stable luciferase production earlier (10–12 days) than the lipofected (13–14 days) with higher signal intensity (295 ± 9.8 and 220 ± 11.3 RLU, respectively). The microscopy data obtained on the 21’th day of the experiment (Fig. S5, Section S8 Supporting Information), revealed similar mCherry^+^ expression for optotransfected and lipofected samples, respectively.

Another crucial parameter is the overall survival of cell monolayers during the clonal selection. As it was noted above, inevitable cell death usually occurs as a result of competing events, namely the formation of selective antibiotic resistance, accompanied by the integration of the pDNA sequence into the host genome; the pDNA escape and/or degradation. The calculated average number of wells which produced stable clones at the end of experiment in the optotransfected with a pulsed laser (relative number of wells with viable cells at the end of the experiment compared to the starting amount of 30 wells) was significantly higher (58 ± 4.3%, or 18 wells) than that for optotransfection with a CW laser (33 ± 3.1% or 10 wells) and for lipofection (29 ± 3%, or 9 wells). The survival of intact cells to the end of experiment was no less than 92 ± 6.8%, with, thus indicating satisfactory grown conditions for such long-lasting cultivation of the cells on the late passages (more than 12, see Fig. 5b).

Given the experimental data on the HeLa cell model, it can be assumed that optotransfection using a pulsed laser provides stable transfection with superior performance compared to the lipofection using LF. The demonstrated results are preliminary and require further studies on an expanded number of cell types to better understand the capabilities and limitations of our optotransfection system for stable transfection, especially for "hard to transfect" cell lines, and primary cells of interest.

### Optoporation of "hard to transfect" cells

Herein, we demonstrate the performance of the developed system for transfection of "hard to transfect" cells, exemplified on the A431 cells and Raw 264.7 cells (Section S9 Supporting Information). It should be emphasized that almost all commercially available protocols for the transfection of adherent cells with lipocation-based or analogous agents can be used efficiently only with an initial monolayer confluence of about 70%^63^. However, the normal state of A431 cells during cultivation is free detachment from the cultural surface while reaching 70–80% confluence. Thus, due to this feature, these cells are ranged to the list of "hard to transfect", and the only way to perform the efficient transfection is to decrease as possible the starting cell monolayer confluence (Fig. 3d). In addition, commercial chemical agents adversely affect cell survival, and it is practically non-available to modify the reagents ratio and experimental conditions without loss of the transfection efficacy.

As soon as the general morphology properties of A431 cells were similar to the HeLa cells, we started optimizing optoporation conditions with the set as default the irradiation regimes selected above as optimal for HeLa cells (see Section ‘The optoporation experimental setup'). Then we conducted serial irradiations with PI as delivered agent with ranging parameters and found the optimal modes for CW laser (energy 25 J, irradiation time 32 s) and pulsed laser (pulse energy 1.7 μJ, duration 150 ns, pulse frequency 10 kHz, scanning speed 0.03 m/s).

**Figure 6:**
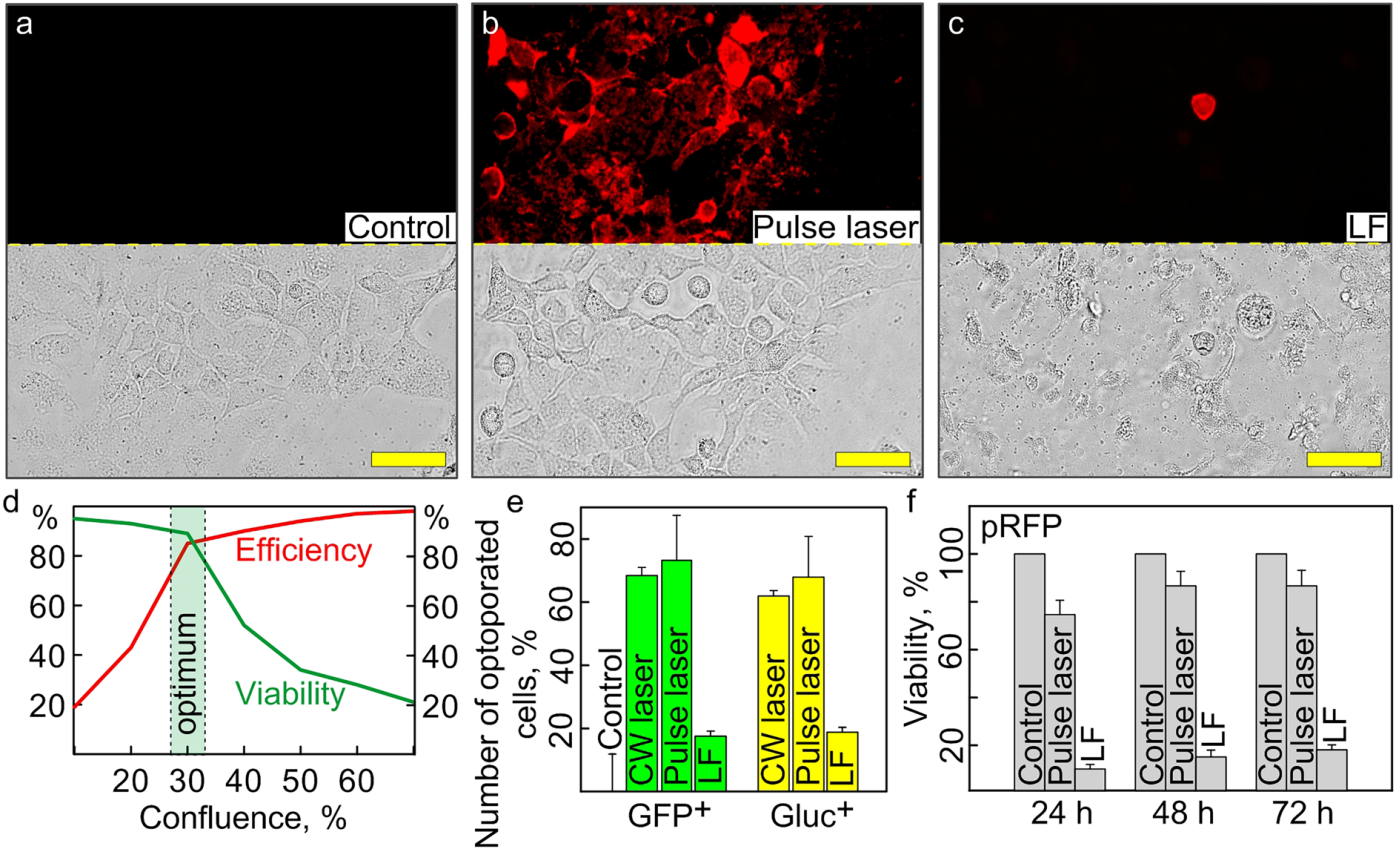
Optotransfection of "hard-to-transfect" A431 cells. (**a-c**) CLSM Microimages of the cells in a fluorescent and phase-contrast mode: (**a**) control cells, (**b)** optoporated cells by CW laser, and (**c**) lipofected cells. (**d**) Determination of the lower threshold starting cell monolayer confluence. (**e**) FACS data: the relative number of pDNA-positive cells optoporated by CW laser and pulsed laser, lipofected cells, normalized to control (intact cells). (**f**) Cell viability data recorded by Alamar blue assay. The scale bars correspond to 50 µm. The mean data is represented in the bars (**e, f**) with the standard deviations at a significance level of *p* ≤ 0.05 set for the error bars.

Then, we investigated the lower threshold of the cell monolayer confluence at the start of the optoporation. For this purpose, the A431 cells were seeded in the wells with AuNS layers with ranging seed dosage 48 h before the experiment to reach starting confluency from 10 to 70% with 10% increments. The wells were subjected to a single optoporation with the pulsed laser under optimized conditions, followed by adding PI dye solution. Then, 30 min post-irradiation, the optoporation efficiency was quantified in terms of the relative number of PI^+^ cells. The cell viability was checked by visual microscopy observation and Alamar blue assay 24 h and 48 h after irradiation (Fig. 6d). As a result, significant inhibition of cell viability was found for the wells with starting confluency less than 30%. Thus, the lower threshold starting confluence for the optotransfection with A431 cells was 30%. Following this algorithm, we estimated the optimal irradiation regimes (pulse energy 1.4 μJ, duration 200 ns, pulse frequency 10 kHz, scanning speed 0.03 m/s) and lower threshold of starting cell confluence (app. 50%), for successful optoporation of Raw 264.7 cells (Fig. S6, Section S9 Supporting Information).

Then, we performed a comparative study of the efficacy of optoporation and lipofection for transient transfection of A431 cells with pDNA. According to the obtained FACS data, the efficiency of optotransfection was significantly higher (72 ± 12% GFP^+^ and 68 ± 9% GLuc^+^ cells) than with LF transfection (18 ± 2% GFP^+^ and 19 ± 1.8% GLuc^+^ cells) (Fig. 6e). It should be noted that LF had a significant cytotoxic effect (14 ± 1.2% according to the Alamar blue assay 72 h after transfection). The viability of optoporated cells under pulsed laser irradiation was maintained at a high level (92 ± 5%) (Fig. 6f). The direct microscopic observation in a phase-contrast mode revealed the good state of the monolayer of optoporated cells (Fig. 6b) similar to the control ones (Fig. 6a). In contrast, for a large number of detached and dead cells presented in the wells with lipofected cells observed (Fig. 6c), thus confirming the FACS and Alamar blue assay data.

In summary, we could transfect "complex" objects with minimal cell monolayer confluence, while commercial agents are designed for only 70% confluency. A pulsed laser provides a wide range of settings for irradiation parameters, which minimizes the risks of cell transfection and significantly increases the efficiency of target agent delivery, with little effect on cell viability. Undoubtedly, one of the main advantages of our plasmonic optotransfection system is the ability to fine-tune the parameters of pulsed laser irradiation to the individual characteristics of objects.

### Optoporation of individual cells

**Figure 7:**
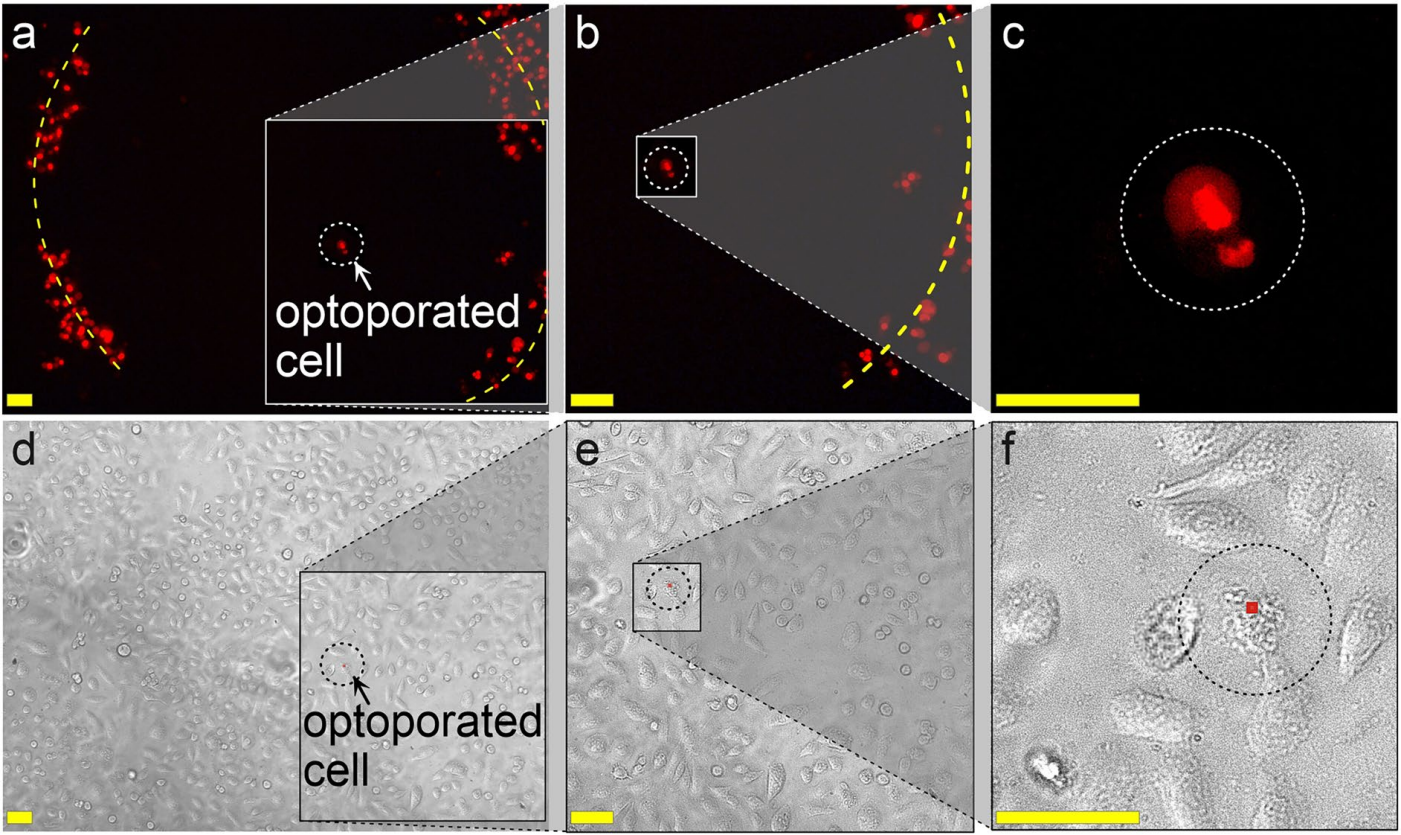
Optoporation of a single cell. (**a-c**) fluorescence microimages and (**d-f**) phase-contrast microimages of CHO cells with a magnification of the single optoporated cell (surrounded by a dotted-line circle); the yellow dotted line shows the trajectory of the pulsed laser for marking the area around the single optoporated cell. The scale bars correspond to 50 µm.

Highly efficient and safe transfection of single cells still remains an important and challenging task. Although recent investigations were performed on this issue^65^, the developed systems have low transfection performance due to several limitations, such as low cell viability due to insufficient optimization and poor scalability. Furthermore, the established plasmonic substrates are fabricated on non-standard cell surfaces, the procedure is rather complexed and need specific equipment. On the contrary, the key beneficial features of our optoporation system which are crucial at the single-cell level, are the following: (1) the optoporation and all following cultivation and visualization steps are performed directly on the well bottoms covered with AuNS layers, in order to minimize any interventions and make the single-cell experiment in a delicate manner; (2) the of irradiation modes within the AuNS layers parameters can be precisely adjusted to the desired cells; (3) the mesh grids on the AuNS layers makes convenient the long-term tracking of the individual cells. Therefore, we performed a proof-of concept experiments exemplified on the PI delivery to the Chinese hamster ovary epithelial cells (CHO) aimed at the demonstration of the listed features. These cells are traditional targets for in vitro single-cell studies due to their ability to form a confluent monolayer from a single cell. As a starting point in the search for optimal cell irradiation modes, the already found irradiation modes for HeLa cells. Specifically, we used a pulse energy of 1.6 µJ, a pulse duration of 200 ns, and a pulse frequency of 10 kHz. At the initial state, we designated the area where the target single cell should be located. For this purpose, we optoporated the cell monolayer area in a circle-way trajectory with a scanning speed of 25 mm/s. Then, we positioned the laser beam in its center with subsequent irradiation without X–Y displacement. As a result, we observed the circle of PI^+^ cells within the single labeled cell in its center (Fig. 7). The XY position of the optoporated cell was fixed using the grid coordinates on the AuNS layers, and then the cell morphology was recorded using direct phase contrast microscopy within 72 h after irradiation with a time period of 6 h.

Notably, the irradiated cells exhibited excellent viability in terms of normal cell division, adhesive properties, and overall morphology, similar to intact cells. Thus, we can conclude that the developed optoporation system in the format of AuNS grid layers prepared on standard multiwell plates can be proposed for efficient and safe transfection of single cells with convenient long-term single cell tracking. Moreover, the developed setup can be useful for further mechanistic investigations. One of the most interesting and not yet exactly clarified queries is related to the current nature of the cell membrane perforations formed upon the plasmonic-assisted irradiation. The recent studies are mainly referred to the heat-shock^39^ or the vapor nanobubbles^40^ concepts, depending on the optoporation regimes. Also debatable are the characteristic life-times, the sizes of membrane perforations, the mechanisms of cell recovery. In our recent study^52^ we explored the cells monolayer behavior and recovery upon irradiation by means of mechanistic properties recorded by life-time atomic-force microscopy. The obtained data were not sufficient to clearly understand the behavior of the system. Therefore, using the present setup at the subcellular resolution, combined with high-throughput analytical tools and approaches, such as cryo-SEM microscopy, cell membrane phantoms, along with simulation instruments, such as COMSOL or similar ones, open up fundamentally new exploration level with wide possibilities.

## Conclusion

In the present work, we have demonstrated the capabilities of the developed optotransfection system based on the Au nanostars layers mediated by resonant NIR laser irradiation. One of the key features of our system is the plasmonic substrates fabricated using the developed centrifugation-based technology directly on the standard culture plates or Petri dishes with the desired well type. The layers can be fabricated with ranging 2-D packing density, with varying plasmonic properties, using different Au nanoparticles if needed, thus fitting for the laser source wavelength. The layers have good batch-to-batch reproducibility, high uniformity, and excellent biocompatibility. The desired size mesh grids on the layers can be applied via the developed laser engraving technique for precise cell experiments. The plates can be fabricated in multiple amounts as needed for a broad experimental set and stored for a long time.

We performed a quantitative comparative analysis of optoporation efficacy versus commercially available lipofection, the summarized data from luciferase assay, CLSM and FACS analysis is given in Table S5, Section S10, Supporting information. We have demonstrated the high level of optotransfection using both laser types on the model HeLa cells with more than 90% of the pDNA transient transfection efficacy and good viability of the irradiated cells. We have obtained HeLa cells with stable expressed fluorescent proteins encoded in the pDNA, by clone selection of transiently transfected cells via optoporation. The average performance of stable transfection was sufficiently better than that for lipofection, as presented in the period for harvesting the full monolayer with cloned cells and the average survival in terms of the well number to the end of the experiment. The experiments with hard-to-transfect cells, namely the A431 line and Raw 264.7 line, under adjusted optimal irradiation conditions, resulted in sufficiently increased transfection efficacy as compared to the lipofection. Notably, the fine adjustment of irradiation regimes fitting to the individual cell parameters is a promising and advantageous feature of our system regarding further issues. Finally, we have demonstrated the optoporation performance at the single-cell level on the CHO cell. The successful optoporation of individually selected irradiated cells has been evidenced by delivered propidium iodide dye molecules and maintained viability for at least 72 h post optoporation.

In summary, the given proof-of-concept study revealed the high performance of the developed optoporation system, which can be proposed as a new tool for precisely controlled, safe, and effective laser transfection compatible with broad types of cells and delivered objects of interest. We believe that our optoporation system based on GNP layers could be helpful in multiple tasks with primary and stem cells in finding novel solutions for personalized gene therapy, regenerative medicine, and other trials based on bioengineered cells.

### Supplementary Information

Supplementary Video 1.

Supplementary Information 1.

Supplementary Information 2.

## Data Availability

The datasets used and/or analyzed during the current study available from the corresponding author on reasonable request.
