# Supplementary material for: High-throughput cell optoporation system based on Au nanoparticle layers mediated by resonant irradiation for precise and controllable gene delivery
Source: Sci Rep. 2024 Feb 6;14:3044. doi: 10.1038/s41598-024-53126-9 (PMC10847436; doi:10.1038/s41598-024-53126-9)
Supplement: Supplementary file 2 — Supplementary Information 1. [file 41598_2024_53126_MOESM2_ESM.pdf]

SUPPORTING INFORMATION

High-throughput cell optoporation system based on Au nanoparticle layers mediated by resonant NIR-irradiation for precise and controllable gene delivery

Pylaev T.E.[1,2,3]*, Avdeeva E.S.[1,2], Khlebtsov B.N.[2], Lomova M.V.[3], Khlebtsov N.G.[2,3]

[1]Saratov Medical State University n.a. V.I. Razumovsky;
410012, Saratov, 112 Ulitsa Bolshaya Kazachya
[2]Institute of Biochemistry and Physiology of Plants and Microorganisms – Subdivision of the Federal State Budgetary Research Institution Saratov Federal Scientific Centre of the Russian Academy of Sciences;
410049, Saratov, 13 Prospect Entuziastov
[3]Saratov National Research State University, 83, 410012, Russia;
410012, Saratov, 83 Ulitsa Astrakhanskaya

*e-mail: pylaev.te@staff.sgmu.ru

CONTENT

Section S1. Reagents
Section S2. Plasmid DNA characteristics
Section S3. UV-vis characterization of AuNS colloid and layer
Section S4. Selection of optimal optoporation regimes for pulsed laser
Section S5. Various types of biomolecules delivered to the HeLa cells using the optoporation system
Section S6. Production of plasmid DNA preparations by molecular cloning
Section S7. Obtaining a HeLa cell line with stable expression of the fluorescent protein gene
Section S8. Optoporation of "hard-to-transfect" Raw 264.7 cells
Section S9. Optotransfection vs lipofection cross-validation study results

Section S1. Reagents

The following reagents were used in the work: chloroauric acid $HAuCl_4$ (Sigma-Aldrich, USA), absolute ethanol C_2H_6O (Scharlau Chemie, Spain, 99.99%), hydrochloric acid HCl (Vecton, Russia), bactotryptone (BD), kanamycin (99%). Poly-4-vinylpyridine (PVP), trisodium citrate dihydrate $C_6H_5O_7Na_3 \times 2H_2O$ (99%), L(+)-ascorbic acid $C_6H_8O_6$ (AA), silver nitrate $AgNO_3$, antibiotic mixture penicillin-streptomycin-neomycin, trypsin, 4,6-diamidino -2-phenylindole $C_{16}H_{15}N_5$ (DAPI), 3-(4,5-dimethylthiazol-2-yl)-2,5-diphenyl-tetrazolium bromide (MTT), resazurin sodium salt $C_{17}H_6NNaO_4$, ampicillin $C_{16}H_{19}N_3O_4S$ (100%), were all purchased from from Sigma-Aldrich with the highest purity. Yeast extract, agar-agar, 2-(N-morpholino)-ethanesulfonic acid $C_6H_{13}NO_4S$ (MES), DL-dithiothreitol $C_4H_{10}O_2S_2$ (DTT) were obtained from Helicon (Russia). Lipofectamine 2000 (LF) transfection reagent was purchased from Fermentas (ThermoFisher Scientific, USA). The QIAamp DNA Mini Kit was manufactured by Qiagen (Germany). The selective antibiotic, geneticin disulfate salt (G418), was purchased from Capricorn (USA). Nutrient medium DMEM, fetal bovine serum (FBS, 10%), Versen's solution, phosphate-buffered saline tablets (PBS, pH 7.4 0.01M) were purchased from Biolot (Russia). Sodium hydroxide NaOH, magnesium chloride $MgCl_2$ (99%), magnesium sulfate $MgSO_4$ (99.79%), glucose $C_6H_{12}O_6$ (99%), potassium chloride KCl (99%) were purchased from Dia-M (Russia). Sodium chloride NaCl (analytical grade), calcium chloride dihydrate

$CaCl_2$*2 H_2O (analytical grade), potassium hydroxide KOH (analytical grade), potassium acetate $C_2H_3KO_2$ (analytical grade) were purchased from EKROS. N,N-dimethylformamide C_3H_7NO (DMF), diethylamine $C_4H_{11}N$ were purchased from AppliChem (USA). Fluorescein diacetate $C_{10}H_{12}N_4O_5$ (FDA) and SlowFade® Gold anti-fading reagent were purchased from Invitrogen (USA). Potassium carbonate K_2CO_3 and dimethyl sulfoxide C_2H_6OS (DMSO) (ACS class) were purchased from Reakhim (Russia). In all experiments, deionized water was used, obtained by the Milli-Q Integral 5 system (Millipore Merck, Germany) with preliminary purification by the Mediana-Filter (Mediana, Russia) system.

Section S2. Plasmid DNA characteristics

We used commercial vectors based on the plasmid DNA carrying genes for fluorescent proteins with emission in a wide spectral range, expressed in the mammalian cells under the control of the CMV promoter. All vectors are equipped with cassettes with antibiotic resistance genes (neomycin) for selective selection in eukaryotic cells. In addition, they contain an additional SV40 promoter and specific antibiotic resistance cassettes (see below) for cloning and selection in prokaryotic cells. The number of copies in bacterial cells is 10-50 copies/cell. Below are the individual characteristics of the vectors (Table S1).

Table S1 – Plasmid DNA used in the work.

Name	Size, b.p.	Molecular weight, MDA	$\lambda_{ex}/\lambda_{em}$, nm	Encoded protein	Type of protein expression	Antibiotic resistance	Manufacturer
pGLuc	5802	1.8	475/570	GLuc	Extracellular	ampicillin	*pCMV-GLuc-2* # N8081, BioLabs (UK)
pGFP	4707	1.4	488/520	GFP	Cytosolic	ampicillin	*phMGFP* #TB320, Promega (USA)
pRFP	4722	1.4	530/650	mCherry	Cytosolic	kanamycin	*pRabCherry* #27679, Addgene (USA)

Section S3. UV-vis characterization of AuNS colloid and layer

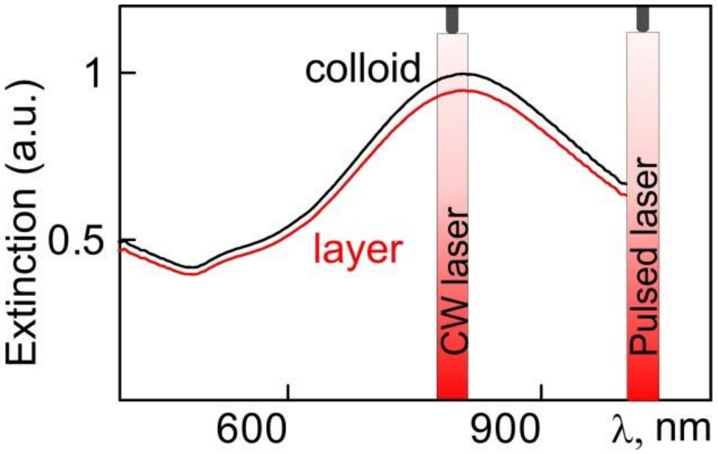

Figure S1 - Normalized extinction spectra of the original freshly synthesized AuNS colloid and the obtained 2-D layers on the surface of the 24-well plate bottom.

Section S4. Selection of optimal optoporation regimes for pulsed laser

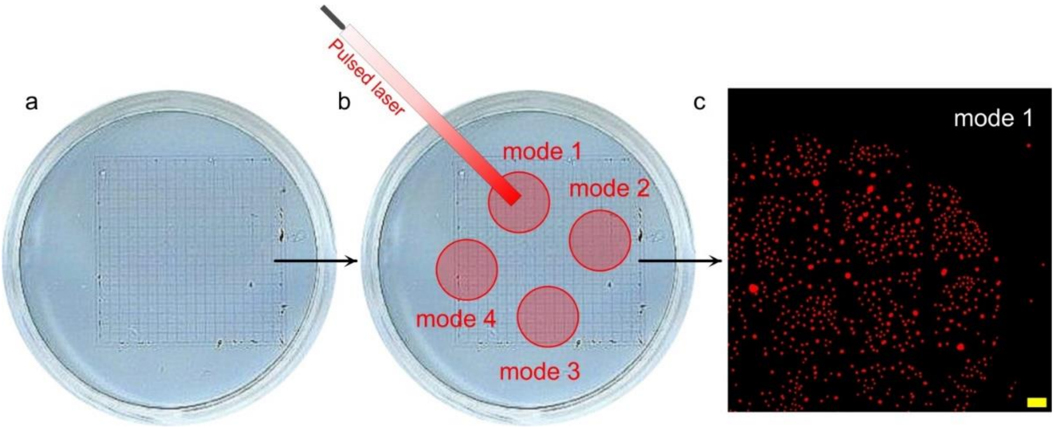

Figure S2 - Illustration of the selection of irradiation modes for pulsed laser: **a** scanned image of the well of a 24-well culture plate with AuNS layer with packing density equal to [Au]~ 28 μg/cm^2 and applied coordinate mesh grid; **b** selection of optimal irradiation modes within one well (mode 1-mode 4); **c** microimage of PI$^+$ cells irradiated in mode 1. The scale bar corresponds to 500 μm.

Section S5. Various types of biomolecules delivered to the HeLa cells using the optoporation system

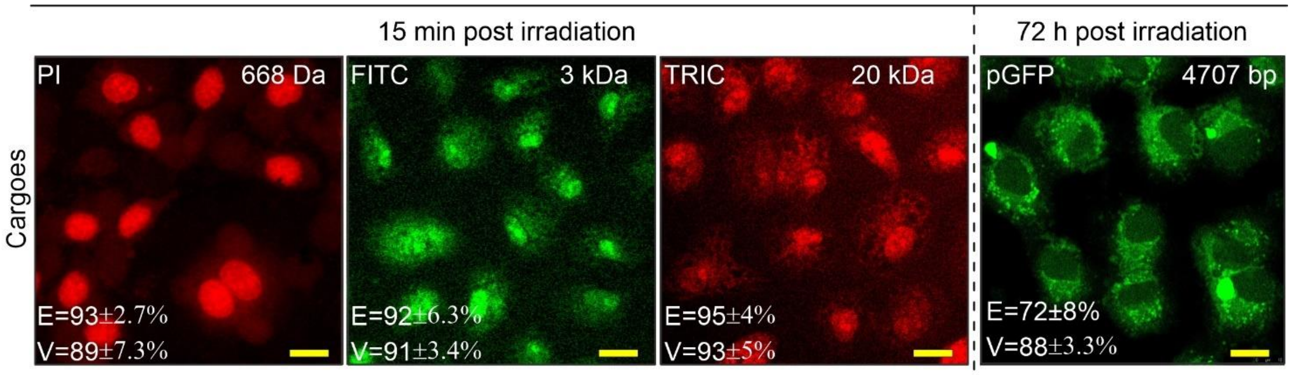

Figure S3 - Optoporated HeLa cells with biomolecules with ranging molecular weights. Microimages of the cells were taken in the fluorescence mode. The scale bars correspond to 20 μm.

Section S6. Production of plasmid DNA preparations by molecular cloning

Bacterial cultures

We used an endonuclease (endA) defective *Escherichia coli* XL1-Blue (no. 632) strain, which was purchased from the collection of rhizospheric microorganisms of the IBPPM RAS.

Cultivation of *E. coli* bacteria, preparation of competent cells, and transformation with DNA plasmids (according to the heat shock mechanism) were carried out according to the generally available methods. [1].

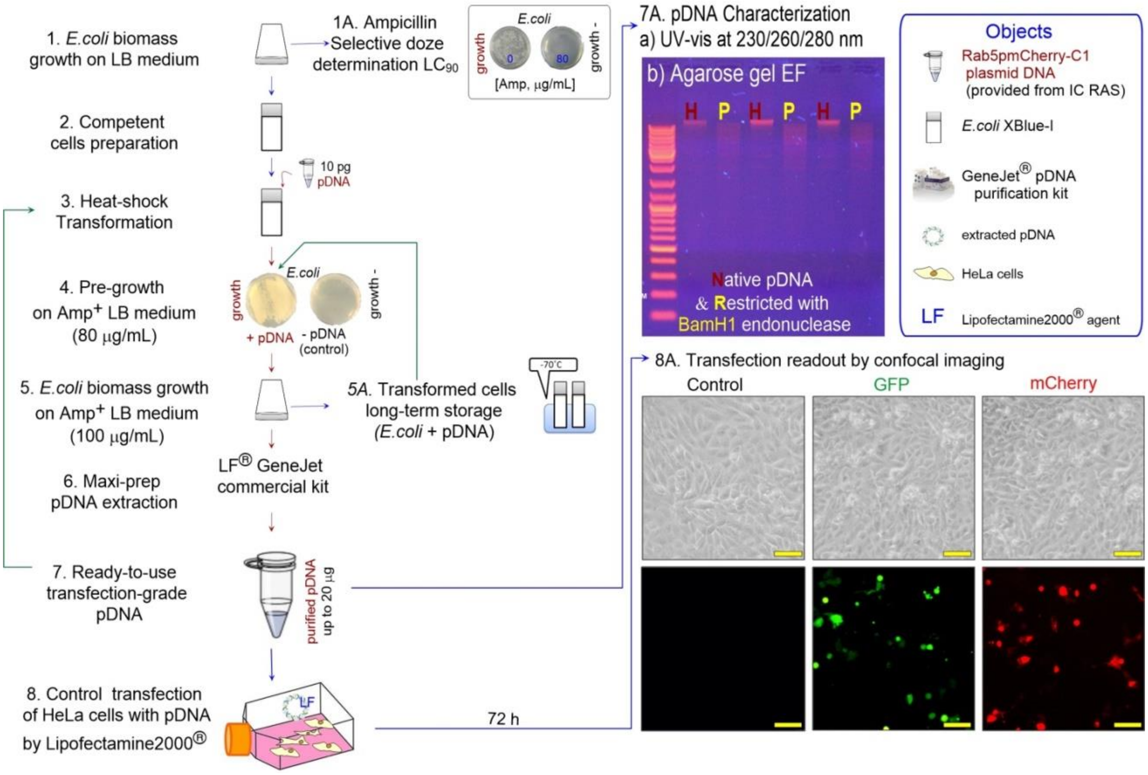

Figure S4 - Step-by-step scheme for the production and purification of pDNA by molecular cloning (1-5) in *E. coli* XL1-Blue cells; restriction analysis (7A) and phase-contrast and fluorescence microimages of HeLa cells, acquired 72 h post control transfection with LF (8). The scale bars correspond to 50 µm.

The selection of transformants and further maintenance of clones was carried out on a solid selective medium LB (Lisogenic Broth) containing 100 µg/ml of the antibiotic. Then, the selected colonies were subcultured onto a liquid selective medium, and plasmid DNA (pDNA) was extracted with the GeneJET Plasmid Midiprep Kit (Thermo Scientifics, USA) according to the manufacturer's recommendations. The isolated pDNA was either used immediately or stored at –20°C.
The amount of highly purified plasmid preparation necessary for the experiments was obtained using the standard cloning technique [2] in *E. coli* XL1-Blue cells, shown in Fig. S4. The obtained transformants containing pDNA were cultured in a liquid nutrient medium, followed by extraction of pDNA in preparative amounts with a commercial plasmid isolation kit, then *E. coli* cells and pDNA preparations were cryopreserved for further use. As a result, pDNA preparations were developed: pGLuc, 650 µg; pGFP, 170 µg; pRFP - 150 µg, purity and quantity were assessed by UV spectrophotometry at the 230/260/280 nm wavelengths. Restriction analysis (Fig. S4-p.7A) and control transfection of HeLa cells via lipofection (Fig. S4-p.8) showed complete agreement between the optical and molecular parameters of the original pDNA samples.

Characterization of plasmid preparations
The plasmid profile was obtained via restriction analysis using commercial enzymes EcoR1 and BamH1 according to the manufacturer's instructions. Briefly, 1 µg of pDNA and 1 µl of restriction enzyme in a supplied buffer were added to the reaction mixture, and the total volume of the mixture was adjusted to 25 µl with Milli-Q water. The reaction tubes were incubated for 5 h at 37°C, then the reaction was inhibited by adding 4 µl of glycerol-containing gel loading buffer to each tube. Restriction products were subjected to electrophoretic separation on a 1% agarose gel prepared in 1X TBE buffer (54 g Tris, 27.5 g boric acid, 146 g EDTA in 1 L distilled water) supplemented with 0.5 µg/ml EtBr. Electrophoresis was carried out for 40–50 min at a field strength of 6 W/cm.

Section S7. Obtaining a HeLa cell line with stable expression of the fluorescent protein gene

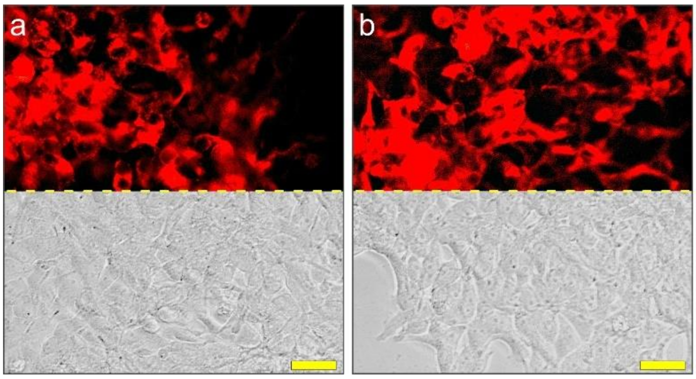

Figure S5 - Fluorescent images and phase-contrast images of HeLa-mCherry^{+} optoporated cells (a) and lipofected (b) cells. The images were acquired at the 21th day after transfection. The scale bars correspond to 50 μm.

Section S8. Optoporation of "hard-to-transfect" Raw 264.7 cells

Optoporation of RAW 264.7 cells, which are monocyte-macrophage-like cells, was performed under the following pulsed laser irradiation modes: pulsed pulse energy 1.4 μJ, pulse duration 200 ns, pulse frequency 10 kHz, scanning speed 25 mm/s. For these cells, the minimum starting monolayer confluence of 50% was found. PI were used as a delivery agent (Fig. S6). Due to the possibility of precise tuning of the irradiation regimes, the PI was successfully delivered to the RAW 264.7 cells, within the high level of the maintained viability above 90% assayed by Alamar-test.

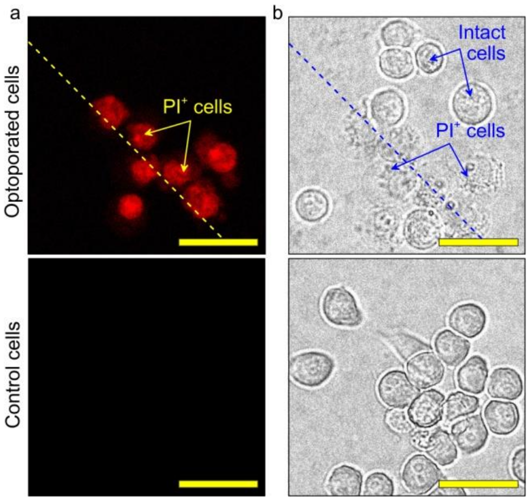

Figure S6 – Optoporation of RAW 264.7 cells. (a) fluorescent images and (b) phase contrast images of the cells; yellow and blue lines show the trajectory of the pulsed laser. The scale bars correspond to 50 μm.

Section S9. Optotransfection vs lipofection cross-validation study results

Summarizing all the obtained data of transient transfection with control pDNA, a summary table S2 is presented below, clearly demonstrating the competitive advantages of the developed original technology of plasmonic optoporation in comparison with commercial lipocation agents.

Table S2 – Cross validation study results of optotransfection vs lipofection for transient transfection of several types cell lines.

Cell type		HeLa	A431	CHO	Raw 264.7
Optimal irradiation modes	E_{imp}, μJ	1.6	1.7	1.6	1.4
	Scanning speed, m/s	0.02	0.03	0.025	0.025
	Pulse duration, ns	200	150	200	200
Optoporation results	Minimum confluence, %	70	30	70	70
	pDNA delivery efficiency, %	72±8*	72±12*	n.a	n.a
	Viability, %	90±4	92±5	83±10	74±9.3
Lipofection data	Minimum confluence, %	70	70	70	70
	pDNA delivery efficiency, %	69±11	18±2	51-79[3]	30[4]
	Viability, %	75±5	14±1.2	n.a	n.a

REFERENCES

1 Glover, D. M. DNA cloning: a practical approach. **1**, 538 (1985).

2 Maniatis, T., Fritsch, E., Sambrook, J. Molecular Cloning. Methods of Genetic Engineering. *World*, 480 (1984).

3 https://www.thermofisher.com/ru/ru/home/technical-resources/cell-lines/c/cell-lines-detail-30.html

4 https://www.thermofisher.com/ru/ru/home/brands/product-brand/lipofectamine/lipofectamine-3000.html#validated
